# Serum levels of the IL-6 family of cytokines predict prognosis in renal cell carcinoma (RCC)

**DOI:** 10.1007/s00262-020-02655-z

**Published:** 2020-07-03

**Authors:** Gigja Gudbrandsdottir, Helene H. Aarstad, Leif Bostad, Karin M. Hjelle, Hans J. Aarstad, Øystein Bruserud, Tor Henrik Anderson Tvedt, Christian Beisland

**Affiliations:** 1 Surgical Clinic, Department of Urology, Haukeland University Hospital, 5021 Bergen, Norway; 2 Department of Pathology, Haukeland University Hospital, Bergen, Norway; 3 Department of Otolaryngology/Head and Neck Surgery, 5021 Bergen, Norway; 4 Department of Clinical Medicine, University of Bergen, 5021 Bergen, Norway; 5 Department of Clinical Science, University of Bergen, 5021 Bergen, Norway; 6 Department of Internal Medicine, Haukeland University Hospital, Bergen, Norway

**Keywords:** IL-6, IL-27, gp130, Survival, Recurrence, Renal cell carcinoma

## Abstract

**Purpose:**

An improved understanding of RCC immunology should shed further light on RCC tumor biology. Our objective was to study to what extent serum levels of the IL-6 family of cytokines at diagnosis were relevant to survival.

**Methods:**

A total of 118 consecutively patients with RCC, in which the tumor was surgically removed at Haukeland University Hospital during the period from 2007 to 2010, were included. The patients were followed-up for 10 years. The morning before surgery blood was sampled and serum frozen, with levels of IL-6, IL-27, IL-31, OSM, CNTF, IL-6Rα and gp130 determined.

**Results:**

Among patients with the highest quartile of IL-6 (> 8 pg/ml) (*n* = 29), six of nine who had metastasis at diagnosis had such high IL-6 values. Among presumed radically treated patients, a high IL-6 and IL-27 strongly predicted recurrence. In particular, the predictions among patients with large (diameter > 7 cm) tumors were excellent regarding both IL-6 and IL-27 values. High gp130 serum levels predicted an overall survival (OS) among RCC patients with large tumors. Patients with a high IL-6 exhibited a strong expression of IL-6 in endothelial- and vascular smooth muscle cells. Moreover, the level of intra-tumoral CD3-positive cells predicted survival.

**Conclusions:**

IL-6 and IL-27 seem to play a role in RCC biology. IL-6 enables the pinpointing of metastatic condition at diagnosis, as well as together with IL-27, the predicting of survival and recurrence. Endothelial cells and vascular smooth muscle cells are both suggested as important sources of IL-6.

**Electronic supplementary material:**

The online version of this article (10.1007/s00262-020-02655-z) contains supplementary material, which is available to authorized users.

## Introduction

Cancer diseases are major global killers of humans [1]; thus, there is an urgent need to better understand these diseases. It is generally accepted that carcinomas are caused by somatic DNA mutations with a consequent dysregulation of the affected cells. Furthermore, it has been known that carcinomas are not only built by actual carcinoma cells, but also, e.g., by intra-tumor immune cells. Biological information from carcinomas is collected with a biopsy, or from a resected tumor, both of which are instant pictures of a long-term ongoing process. One important source of tumor biology knowledge is serum samples, of which tumor-associated secretory interleukins/cytokines contribute, with the study of this cytokine reservoir in cancer patients being the primary goal of this study. One primary validity criteria of all cancer studies is the association to prognosis, so we will therefore presently use survival as our readout variable.

Renal cell cancer (RCC) is the ninth most common cancer in men and 14th most common cancer in women. In 2018, 175,098 deaths by RCC were estimated, making it the 14th most common cause of global cancer deaths [2]. RCC represents one of the major immunogenic carcinomas [3]. Over the last few years, biological therapy has gained importance as a treatment for metastatic RCC, mostly by VEGF blockage [4]. Recently, modern immune therapy has also been introduced [5].

IL-6 is a cytokine produced by, e.g., macrophages, Th2 cells, B cells, astrocytes, endothelial cells, adipocytes and some tumor cells [6]. IL-6 has been shown to promote tumor proliferation, metastases and symptoms of cachexia [6]. In a review paper, the IL-6 serum level at diagnosis was significantly correlated to survival in 82/101 series, comprising 9917 out of 11,583 patients with 23 different cancer types [7].

IL-6 regulates inflammation by two main pathways: The *classic* signaling, in which it binds to a membrane-bound IL-6α receptor expressed in only a few cell types and then secondarily to membrane-bound gp130 (signal transducing receptor glycoprotein 130 kDa) present in many cells [8]. The *trans* signaling IL-6 binds to membrane gp130 through a primary binding to serum IL-6Rα [9]. The *classical* signaling stimulates the regenerative and anti-inflammatory activity, whereas *trans* signaling has more general stimulatory effects [9]. When IL-6/IL-6Rα binds to gp130, three signaling pathways may be activated: JAK-STAT, Ras-ERK cascade or P13K-Akt signaling. Through all three ways of *trans* signaling, IL-6 promotes the growth of cancer cells, whereas STAT3 IL-6 also promotes tumor cells’ ability to escape apoptosis [10]. On the other hand, soluble gp130 receptor serves as a decoy receptor that inhibits the function of IL-6/IL-6Rα complex [11].

Several other cytokines also share the use of gp130 subunit receptor. These cytokines are collectively named the IL-6 family of cytokines [8, 12], which has several members, including IL-11, IL-27, IL-31, ciliary neurotrophic factor (CNTF), leukemia inhibitory factor (LIF), oncostatin M (OSM) and cardiotrophin-like cytokine factor 1 (CLC) [13]. The receptor signaling complexes for IL-6 and IL-11 contain a gp130 homodimer, whereas other family members signal via a heterodimeric receptor complex containing gp130 [13].

IL-6 has been shown to be secreted from RCC cells exposed to hypoxia, and hypothesized to contribute to RCC invasion and the development of metastasis [14–16]. In RCC serum IL-6 levels have been associated with extended tumor stage, grade and metastatic progression [16].

Regarding other IL-6 family cytokines, Pu et al. [17] showed that two polymorphisms in the IL-27 gene were associated with an increased risk for RCC. IL-27 acts through a receptor consisting of IL-27Rα and gp130, which mediates signaling mostly through STAT1 and STAT3, though similarly to IL-6. IL27-Rα is present on B, T and NK cells, neutrophils, monocytes and mast cells, as well as in lower levels in macrophages, hepatocytes, keratinocytes and endothelial cells [18]. IL-27 has demonstrated antitumor activity in prostate cancer, multiple myeloma, non-small cell lung cancer and ovarian cancer cell lines [18]. In contrast, high serum levels of IL-27 in breast and gastroesophageal cancer are correlated with advanced stage [18].

Tumor diameter, measured by CT prior to surgery, is a strong indicator for survival [19]. Most RCC-caused deaths occur in patients with tumors > 7 cm in diameter. Hence, we have aimed in particular at studying large RCC tumors as to evidence for cytokine involvement.

It is also of interest to study tumor tissue, both for the source of secretion and as a potential target [20]. We have therefore studied the level of macrophages and T lymphocytes in and around tumors, as well as IL-6 and IL-6 receptor levels on endothelial cells, macrophages and T lymphocytes, both in and adjacent to the tumors in patients with a high serum IL-6.

In this study, we have aimed at investigating whether the IL-6 family of cytokine members and pertinent cytokines receptors levels, both in serum preoperatively and in tumor tissue, relate to RCC biology by studying the prognostic value of these cytokine/receptor levels at diagnosis.

## Material and methods

### Patients

From the kidney cancer database at Haukeland University Hospital, we identified 159 consecutive patients treated with nephron sparing surgery (NSS), a radical nephrectomy (RN) or a cyto-reductive nephrectomy at our institution between January 1, 2007 and March 31, 2010. All histological subtypes and stages were included. For IL-6 analyses, 118 patients with appropriate blood samples were available, while for the other cytokine analyses 97 patients were available. Attrition analyses revealed no difference in regard to descriptive statistics between individuals registered in the database who had blood samples bio-banked and those who did not. Most patients were male [*n* = 88 (75%)], the mean age was 63 years (median 64, IQR 55–73) and the mean tumor size was 6.3 cm (median 5.3, interquartile range IQR 3.7–8.7). A radical nephrectomy was performed in 66% (*n* = 75) of the patients.

All patients were followed-up to January 18, 2018/10 years or time of death, and the information registered. The follow-up flow chart at Haukeland University Hospital, which is based on Leibovich score (stage, lymph nodes, tumor size, nuclear grade and tumor necrosis) has been previously reported [21]. The mean observation time was 99 (median 105, IQR 95–120) months. During the observation period, 20 patients died from RCC, while 19 patients died from other causes. A total of 14 patients (12%), presumed radically treated, developed metastases during the follow-up period. Our institutional Follow-up regime has been described in detail by our group [22]. The Regional Committee for Medical Research Ethics in Western Norway (78/05), the study and the Norwegian Social Science Data Services all approved the database. All patients signed informed consent forms at inclusion.

### Tumor assessment

Patients were staged according to the 2009 TNM classification system, and the tumor histology was graded according to the Fuhrman nuclear criteria [23].

### Laboratory cytokine assessment

Preoperative blood samples were drawn on the morning of the surgery, and serum was frozen at − 80/150 °C until analysis. Serum IL-6 was detected using the Luminex immune-bead technology and a high-sensitivity kit (Invitrogen/Biosource, Carlsbad, CA, USA). In short, antibody-coupled beads were incubated with serum and incubated with a biotinylated detection antibody, before finally being incubated with streptavidin–phycoerythrin. Samples were then read by the Luminex's laser-based fluorescent analytical test instrument Luminex^®^ 100™ (Luminex Corporation Austin, TX, USA). Gp130, IL-27, IL-31, IL-6Rα, OSM, and CNTF measured with the same method: Human Premixed Multi-Analyte Kit from R&D system, and the latter by the use of the Milliplex map kit Human Pituitary Magnetic Bead Panel 1 (Millipore, Sigma-Aldrich, Oslo, Norway).

### Immunohistological assessment

Tumor tissue from patients with the highest IL-6 serum levels (*n* = 29) was investigated, with one representative block selected from each case. The selected slide contained both tumor tissue corresponding to the tumor ISUP grade and an area bordering on and comprising kidney parenchyma (interphase zone). An experienced uropathologist classified all the RCCs based on hematoxylin and eosin-stained sections.

Immunohistochemistry was performed using the automated benchmark ultra-system (Ventana-Diagnostics Roche). Four-micrometer sections from the formalin-fixed paraffin embedded (FFPE) tissue blocks were de-paraffinized and rehydrated, while antigen retrieval was done by conditioning the cells in a TRIS-based buffer (CC1, Ventana) and heating. After endogenous peroxidase blocking, the slides were incubated with the primary antibodies. Detection was performed by OptiView^®^ (OV) and UltraView^®^ (UV) DAB detection kits (Ventana Medical Systems), with hematoxylin used as a counterstain. Human spleen and lymph node sections were used as positive controls, while for negative controls, primary antibodies were omitted (Supplementary Table 1).

The whole tumor area in the slide was examined and the subjective impression of density and number of positive cells were scored semi-quantitatively and subjectively. The proportion of IL-6 and IL6R-positive tumor cells were scored as “no positive tumor cells” (0), “less than 10% positive tumor cells” (1 +), “10–50% positive tumor cells” (2 +), or “more than 50% positive tumor cells” (3 +). For CD3, CD68 and FOXP3, 1 + means slight and scattered infiltration, 2 + moderate infiltration and 3 + the dense infiltration of positive cells in more than 50% of the area.

### Statistical analysis

Comparisons between groups were performed with cross-tables and exact Chi-square test, Mann–Whitney *U* test and *T* test for categorical, ordinal and continuous data, respectively. A patient’s serum levels of IL-6 ≥ 8 pg/ml (the uppermost quartile), and of IL-27 for the uppermost quartile, were defined as high. The multiple logistic regression models were performed in a backward likelihood ratio (LR) test manner without a pre-selection of the variables.

Kaplan–Meier analyses were used to estimate DSS and recurrence-free survival (RFS). For a survival comparison between different groups, a log rank test was used. A Cox proportional hazard model was used to determine DSS and RFS predictions after adjusting for other variables affecting survival in univariate analyses. Correlations between variables were calculated using Kendall analyses, while ROC curves were used to calculate predictive value, sensitivity and specificity of IL-6/IL-27 as to recurrence. For all statistical analyses, a *p* value of less than 0.05 was considered statistically significant, and calculations were performed using the IBM^®^ SPSS^®^ Statistics software (Release 24.0).

## Results

### IL-6 family cytokines versus tumor characteristics in patients presumed radically treated

**Fig. 1:**
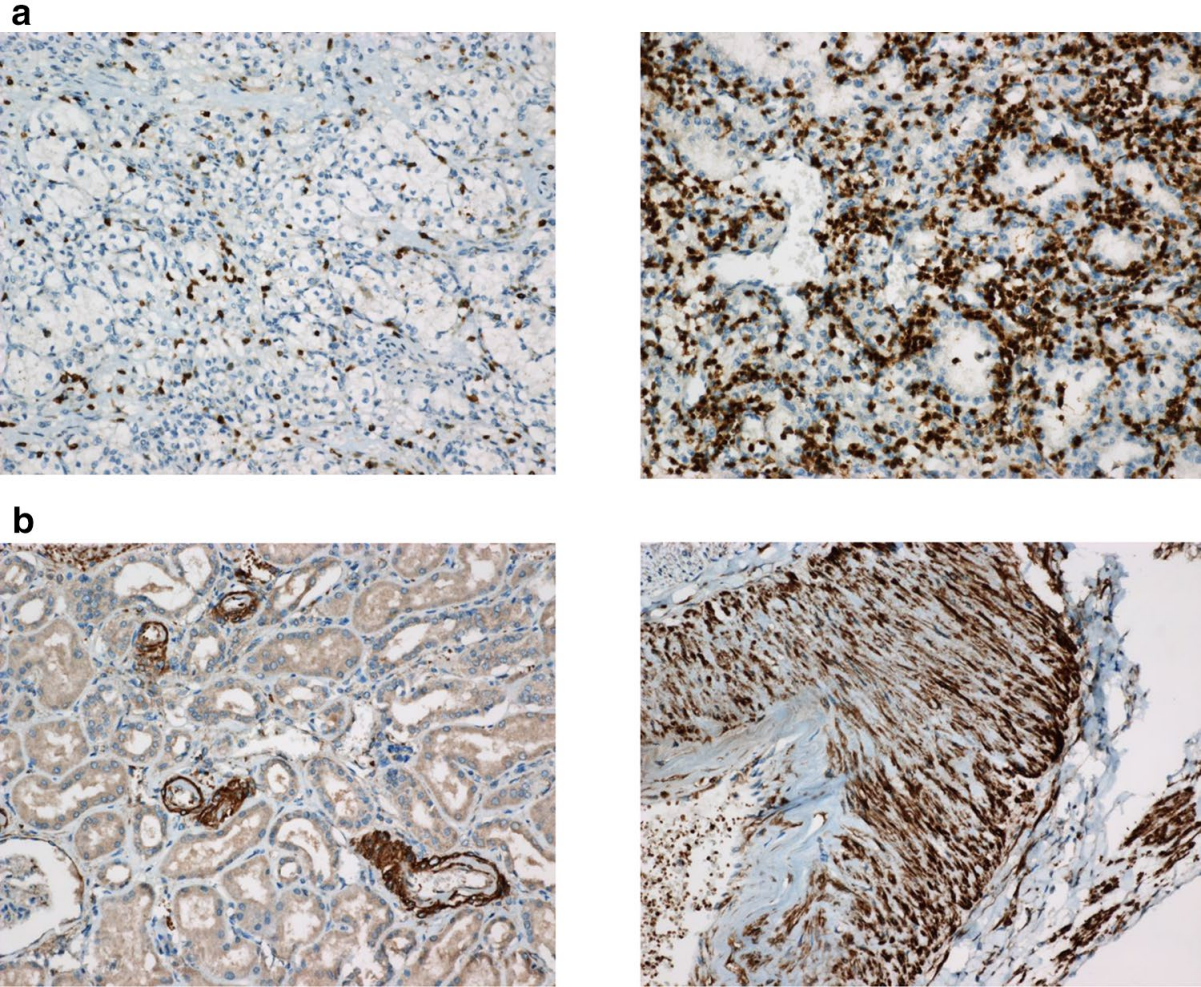
**a** The panels show low (left) and high (right) scores with tumor area CD3 staining lymphocytes. **b** The panels show renal tissue outside the tumor: to the left small arteries showing IL-6 positivity, and to the right interlobular artery showing a strong IL-6 expression in medial smooth muscle cells

The patients (*n* = 109) were divided into two groups, those with a low (IL-6 < 8 pg/ml) vs. high (IL-6 ≥ 8 pg/ml) IL-6 values at diagnosis. The groups did not differ in RCC subtype, tumor size, pathological stage, nuclear grade or other known predictive factors. Histological positive margins (*p* = 0.05) and pT stage (*p* = 0.054) differences were borderline differentiating between the patient groups (Supplementary Table 2). Immunohistochemistry was done in patients with high Il-6 in serum (Fig. 1).

### IL-6 family cytokines and soluble receptors recurrence prediction

**Fig. 2:**
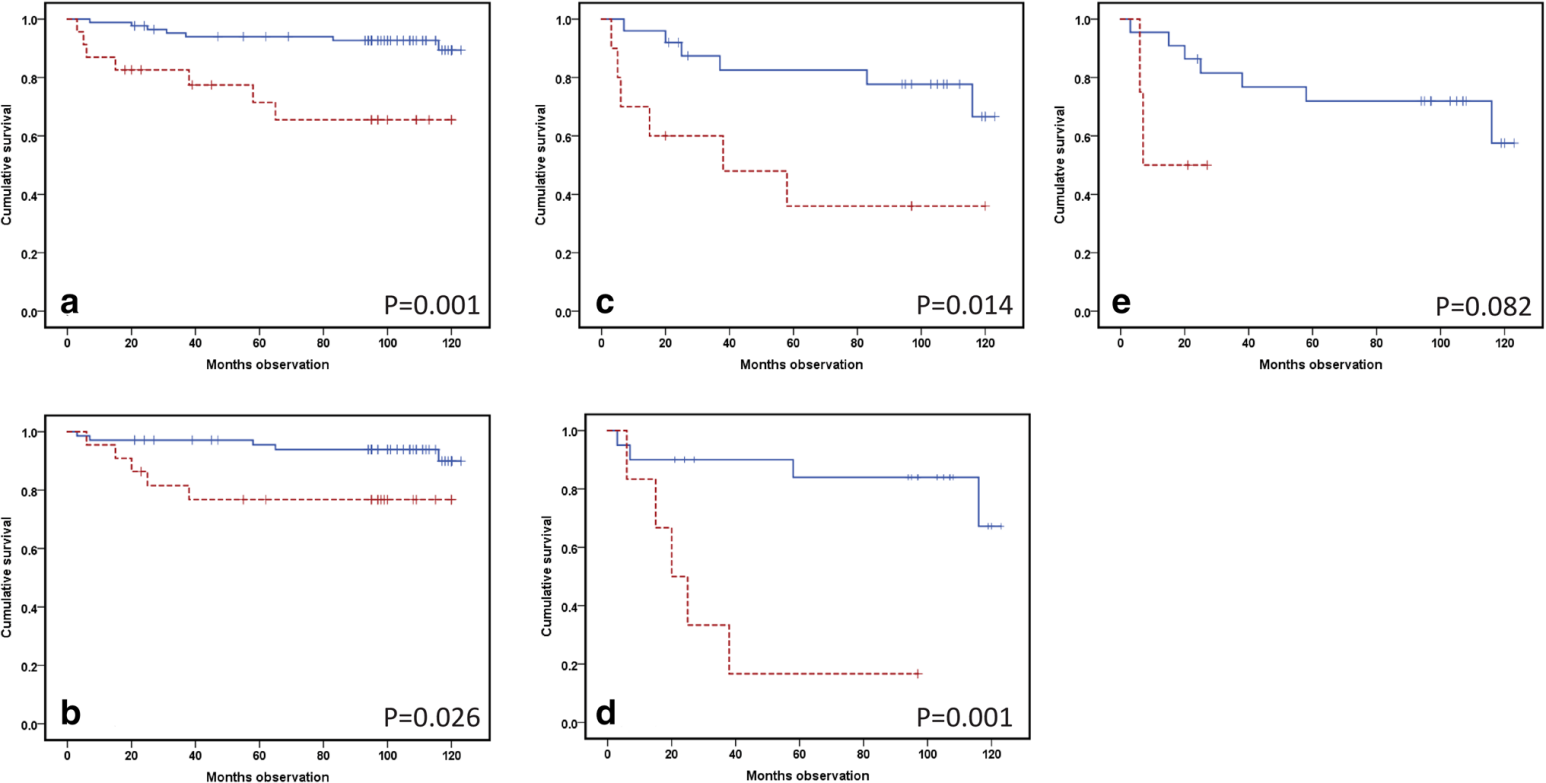
Kaplan–Meier recurrence curves from IL-6 and some family members, as analyzed by Luminex in the serum of assumed radically treated renal cell carcinoma (RCC) patients, sampled prior to surgery. The blue line represents a low value, whereas the red dotted line indicates a high value. Differences between the groups are examined in log-rank tests and presented with *p* values. **a** IL-6 recurrence prediction among 109 RCC patients (low (< 8 pg/ml): *n* = 86 and high (≥ 8 pg/ml): *n* = 23). **b** IL-27 prediction of recurrence in 91 RCC patients (low: *n* = 69 and high: *n* = 22). **c**–**e** Recurrence prediction of IL-6, IL-27, and gp130 in patients with large (> 7 cm) RCC tumors (low: three lower quartiles)/high: highest quartile). **c** IL-6: *n* = 35 (25/10). **d** IL-27: *n* = 26 (20/6). **e** gp130: *n* = 26 (22/4)

IL-6 levels predicted recurrence, both by Kaplan–Meier survival analysis (*p* = 0.001) (Fig. 2a) and utilizing a Cox multivariate regression analysis, with age, gender and tumor size additionally included as covariates (HR 7.13, CI 2.23–22.8; *p* = 0.001) (Table 1). IL-27 showed a significant prediction of recurrence, analyzed by Kaplan–Meier analysis (*p* = 0.026) (Fig. 2b) and multivariate Cox regression analysis, with covariates being age, gender and tumor size (HR 6.89; CI 1.56–30.4; *p* = 0.011) (Table 1).

**Table 1:** Recurrence and survival predictions from IL-6 and IL-27 in Cox regression analyses

	Univariate				Multivariate including age, gender and tumor size			
	HR	95% CI		*p* value	HR	95% CI		*p* value
		Lower	Upper			Lower	Upper	
Recurrence in presumed cured patients								
IL-6, *n* = 109	4.99	1.74	14.3	0.003	7.13	2.23	22.8	0.001
IL-27, *n* = 91	3.77	1.08	13.2	0.038	6.89	1.56	30.4	0.011
Disease-specific survival in all included patients								
IL-6, *n* = 118	4.97	2.06	12.0	< 0.001	4.82	1.96	11.9	0.001
IL-27, *n* = 97	2.82	0.95	8.40	0.062	3.02	0.94	9.64	0.063
Overall survival of all included patients								
IL-6, *n* = 118	2.81	1.46	5.40	0.002	2.99	1.54	5.81	0.001
IL-27, *n* = 97	2.05	0.94	4.50	0.072	1.98	0.86	4.57	0.108

*HR* hazard ratio, *CI* confidence interval

If both IL-6 and IL-27 were included to one Cox multivariate regression analysis, the recurrence of those presumably cured was predicted by IL-6 (*p* = 0.004), but not regarding IL-27 (*p* = 0.082) (Table 2).

**Table 2:** Outcome predictions from combined IL-6 and family cytokine members in Cox regression analyses

	Multivariate (only cytokine/receptor combined)				Multivariate including age, gender and tumor size			
	HR	95% CI		*p* value	HR	95% CI		*p* value
		Lower	Upper			Lower	Upper	
Recurrence in radically treated patients (*n* = 91)								
IL-6	6.64	1.80	24.4	0.004	25.5	3.04	213.4	0.003
IL-27	3.10	0.87	11.1	0.082	1.54	0.20	11.7	0.675
Disease-specific survival (*n* = 97)								
IL-6	7.47	2.26	24.7	0.001	10.8	2.62	44.4	0.003
IL-27	1.98	0.65	6.00	0.227	0.85	0.22	3.30	0.813
IL-6	10.1	3.07	33.4	< 0.001	20.7	5.25	81.4	< 0.001
IL-6Rα	0.17	0.021	1.31	0.089	0.068	0.007	0.66	0.020
gp130	1.11	0.24	5.12	0.889	2.64	0.45	15.4	0.281

*HR* hazard ratio, *CI* confidence interval

If the patients were grouped by tumor size (± 7 cm) and studied by Kaplan–Meier analyses, both IL-6 (*p* = 0.014) (Fig. 2c) and IL-27 (*p* = 0.001) (Fig. 2d) predicted recurrence among patients with large tumors (diameter > 7.0 cm). Regarding gp130 levels there was not a statistic significance (*p* = 0.082) (Fig. 2e).

### IL-6 family cytokines and soluble receptors vs. DSS

**Fig. 3:**
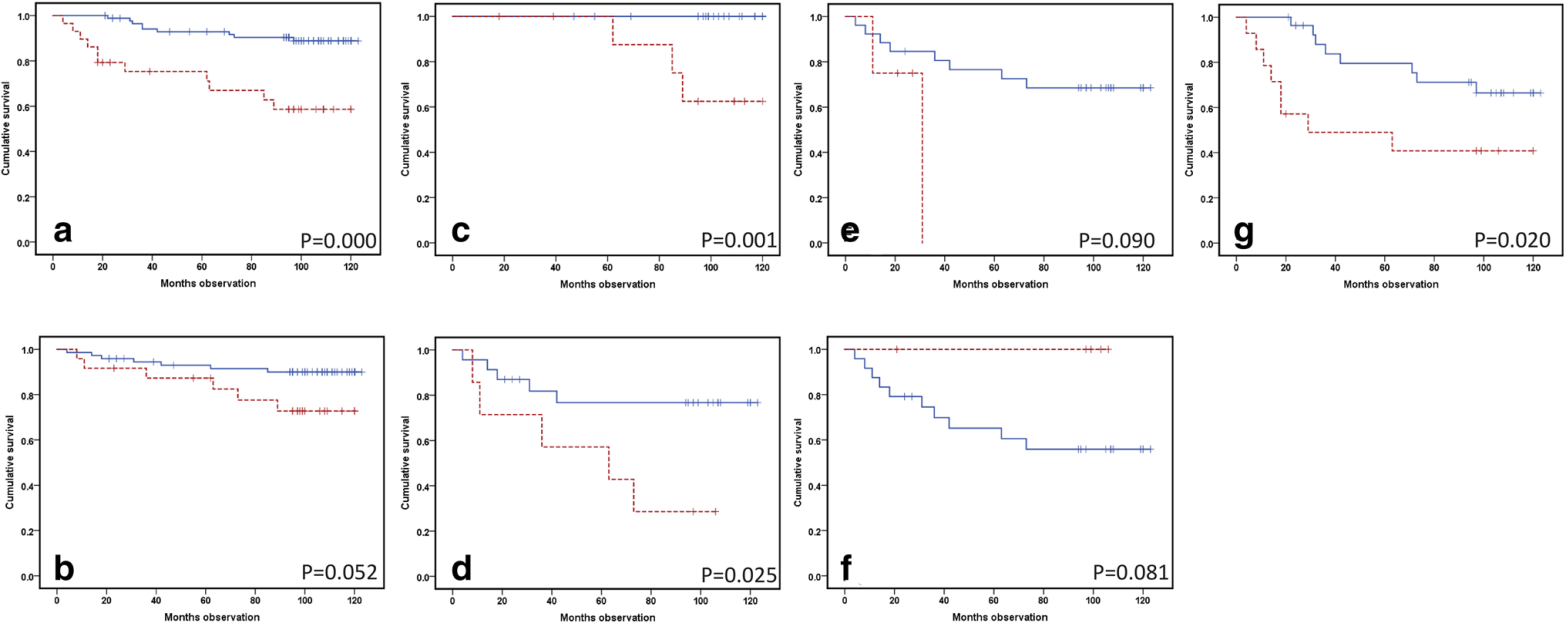
Kaplan–Meier curves demonstrating disease-specific survival (DSS) prediction from IL-6, as well as related cytokines and receptors in pre-treatment RCC blood samples collected before surgical treatment. Analytes were measured simultaneously by Luminex technology. Low values are shown with a blue continuous line, and high values in red dotted lines. In addition, the graphs comprise *p* values from log-rank tests. **a** IL-6 DSS prediction among 118 RCC patients (low (< 8 pg/ml): *n* = 89 and high (≥ 8 pg/ml): *n* = 29). **b** IL-27 prediction of DSS in 97 RCC patients (low: *n* = 73 and high: *n* = 24). **c** and **g** DSS prediction from IL-6 in medium-sized (4.1–7 cm, *n* = 37) and large (> 7 cm, *n* = 42) RCC tumors. The highest quartile is denoted by high (*n* = 10/14), and the remaining values low (*n* = 27/23). **d**–**f** IL-27, gp130 and IL6R alpha prediction of DSS in patients harboring a large (> 7 cm) RCC tumor (*n* = 30). Quartiled analytes as above (high: *n* = 7/4/6 and low: *n* = 23/26/24)

IL-6 predicted DSS in both Kaplan–Meier analysis (*p* < 0.001) (Fig. 3a) and multivariate regression analysis, including gender, age and tumor size (HR 4.82; CI 1.96–11.86; *p* = 0.001) (Table 1). In regard to IL-27, there was a borderline DSS prediction in the Kaplan–Meier analysis (*p* = 0.052) (Fig. 3b).

If both IL-6 and IL-27 were included in one Cox multivariate regression analysis for DSS, only IL-6 levels were predicted (HR 20.7; CI 2.6–44.4; *p* = 0.001) (Table 2).

IL-6, gp130 and IL-6Rα were included to one DSS multivariate analysis that also included gender, age and tumor size in one Cox regression survival model. Subsequently, IL-6 (*p* < 0.001) and IL-6Rα (*p* = 0.02), but not gp130, showed survival prediction (Table 2).

If analyzed by tumor size, patients with a tumor diameter from a 4 to 7 cm IL-6 level predicted survival by Kaplan–Meier analysis (*p* = 0.001) (Fig. 3c). The same was the case with large tumors (tumor diameter > 7.0 cm) (*p* = 0.02) (Fig. 3g). When IL-27 levels were analyzed by size, it was determined that a survival prediction was found among the patients with large tumors (diameter > 7 cm) (*p* = 0.025) (Fig. 3d). Including only tumors > 7 cm, s-gp130 levels exhibited no survival prediction (*p* = 0.09) (Fig. 3e). The same was the case with the soluble sIL-6Rα levels (*p* = 0.08) (Fig. 3f).

### IL-6 family cytokines and soluble receptors vs. overall survival (OS) with all patients included

**Fig. 4:**
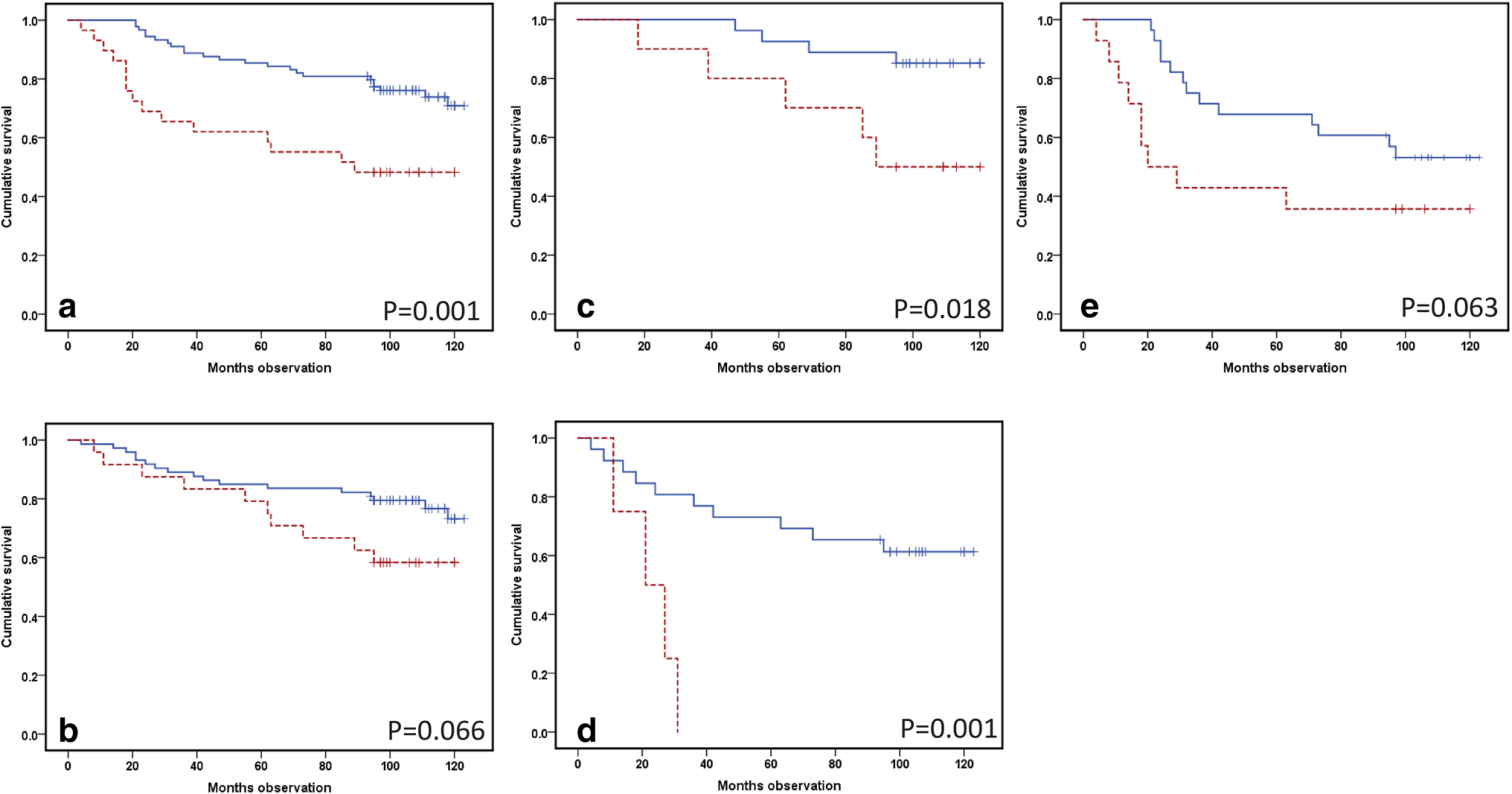
Overall survival (OS) Kaplan–Meier curve predictions from IL-6 and the family molecules IL-27 and gp130 by Luminex in serum collected before the surgical treatment of RCC patients. The blue continuous line visualizes low values (lower quartiles), with the high values in the red dotted line. Log-rank test *p* values are included in the separate windows. **a** IL-6 OS prediction among 118 RCC patients (low (< 8 pg/ml): *n* = 89 and high (≥ 8 pg/ml): *n* = 29). **b** IL-27 prediction of OS in 97 RCC patients (low: *n* = 73 and high: *n* = 24). **c** OS prediction from IL-6 in medium-sized [(4.1–7 cm, *n* = 37), *n* = 42] RCCs. Twenty-seven patients were designated as low, with 10 patients having a value within the highest quartile. **d** Prediction of OS from quartiled gp130 in RCC patients with a tumor size exceeding 7 cm (low: *n* = 26 and high: *n* = 4). **e** IL-6 OS prediction in large (> 7 cm, *n* = 42) RCC tumors; 28 high value patients and 14 with a low value

In Kaplan–Meier analysis, IL-6 values predicted OS (*p* = 0.001) (Fig. 4a). In a Cox multivariate survival analysis, including the gender, age and tumor size of the patient, a significant survival prediction was still determined (HR 2.99; CI 1.5–5.81; *p* = 0.002) (Table 1). IL-27 showed no survival prediction with a Kaplan–Meier approach (*p* = 0.066) (Fig. 4b). Regarding Cox multivariate regression analysis (HR 1.98; CI 0.86–4.57; *p* = 0.11) (Table 1), OS was not predicted. The model was tested and was stable for HR with regard to the IL-6 and IL-27 groups.

If the patients were grouped by tumor size, the IL-6 values in particular predicted survival among patients with medium-sized tumors (tumor diameter from 4 to 7 cm) (*p* = 0.018) (Fig. 4c), but not statistically significant (*p* = 0.063) among large tumors (Fig. 4e). If gp130 levels were studied in patients with large tumors only, a high gp130 level predicted a lower survival (*p* = 0.001) (Fig. 4d).

### Outcome dependent on IL-6 levels at the individual patient level

We detected IL-6 > 8 pg/ml in 29 patients: six of those with metastasis at the time of diagnosis, with seven of the remaining 23 patients presumed radically treated having had a subsequent RCC recurrence. Of those patients with a low IL-6 who died, or developed recurrent RCC disease (*n* = 10), only one had a RCC tumor < 7 cm at diagnosis. Five of the 10 patients with a high IL-6 who were still alive and without disease recurrence at the study closure, had either a second primary cancer or an autoimmune disease at diagnosis.

### Outcome by ROC analyses

**Fig. 5:**
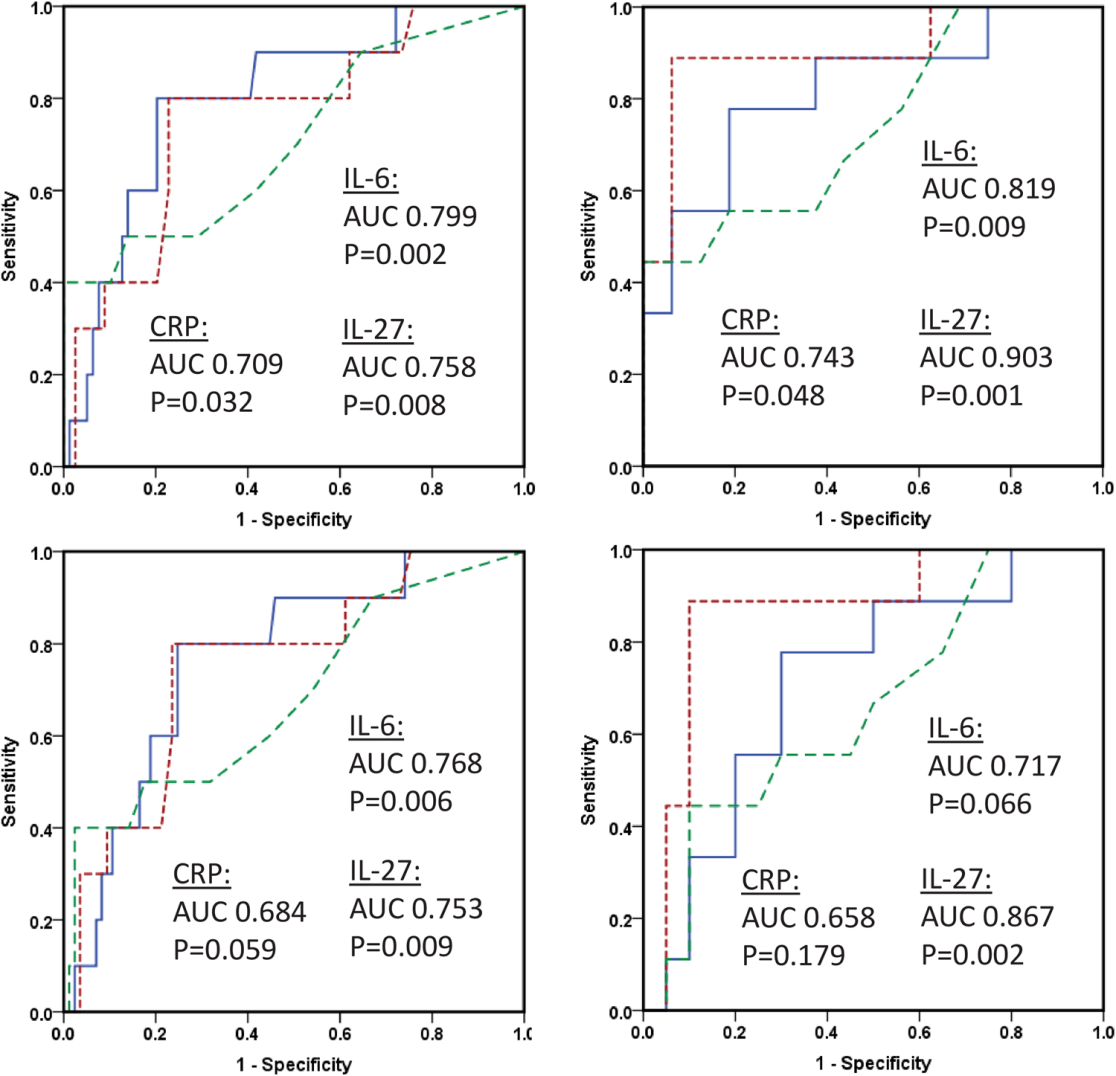
ROC recurrence. Receiver operating characteristic (ROC) curves comparing recurrence prediction of IL-6, IL-27, and CRP in presumed radically treated (upper panel, *n* = 89) and all (lower panel, *n* = 95) RCC patients with such values, as analyzed in their serum ahead of surgical treatment. In both cases, 10 were regarded as positive. The right column shows results in patients with a tumor above 7 cm, of which nine had a positive annotation. The blue continuous line represents IL-6, the red dotted line is IL-27, and green semi-hatched line indicated by CRP

Both tumor diameter and IL-6 values predicted DSS and recurrence. According to IL-6 for recurrence, estimated areas under the curve (AUC) were 0.723 ± 0.075 (*p* = 0.007) and 0.692 ± 0.074 (*p* = 0.020), employing presumed radically treated or all patients, respectively. Regarding IL-27, the corresponding AUC results were 0.762 ± 0.080 (*p* = 0.007) and 0.757 ± 0.079 (*p* = 0.008), respectively (Fig. 5). Including only presumed radically treated patients and with large tumors (diameter > 7 cm), the AUC were 0.908 ± 0.069 (*p* = 0.001) in the case of IL-27, and 0.707 ± 0.098 (*p* = 0.048) in the case of IL-6 (Fig. 5).

### IL-6 and IL-27 prediction of recurrence adjusted by Leibovich scores

IL-6 and IL-27 levels were studied by Cox regression adjusted by Leibovich scores. The results showed that both IL-6 (*p* = 0.01) and IL-27 (*p* = 0.014) still predicted survival following such an adjustment (Supplementary Table 3).

### Patients with high serum IL-6: outcome compared to tumor and tumor border (interphase) tissue CD3, CD68, IL-6 and IL6R-positive cells determined by immunohistochemistry

By immunohistochemistry, we have determined the level of CD3, FoxP3, CD68, IL-6 and IL6R-positive cells in tumors from patients with high IL-6 serum values (Table 3). The following number of patients with at least a 10% (1 +) expression on markers denoting cell *characteristics* were found at least at 1 + levels: intra-tumor CD3 + lymphocytes: 22/28, interphase zone CD3 + lymphocytes: 18/28, intra-tumor CD68 + cells: 25/28, interphase zone CD68 + cells: 8/28, FoxP3 + intra-tumor lymphocytes: 2/27, FoxP3 + interphase zone lymphocytes: 4/27 and FoxP3 + tumor cells: 0/27.

**Table 3:** Description of immunohistochemical analyses, staining assessment and numbers

	– = 0.0	± = 0.25	± = 0.5	+ = 1.0	+ (+) = 1.5	++ = 2.0	++ (+) = 2.5	+++ = 3.0
CD3-positive tumor lymphocytes	1	0	5	11	4	2	4	1
CD3-positive lymphocytes in interphase zone	3	0	7	10	2	4	1	1
CD68-positive cells in tumor	0	0	3	7	7	6	5	0
CD68-positive interphase zone cells	14	1	5	5	1	2	0	0
FoxP3 in tumor lymphocytes^a^	9	14	2	2	0	0	0	0
FoxP3 in interphase zone lymphocytes^a^	9	8	6	3	0	1	0	0
FoxP3 in tumor cells^a^	25	2	0	0	0	0	0	0
IL6 in tumor lymphocytes	18	2	4	4	0	0	0	0
IL6 in interphase zone lymphocytes	16	10	2	0	0	0	0	0
IL6 in tumor cells	5	8	6	4	2	3	0	0
IL6 in vasculature	1	2	1	3	2	6	1	12
IL6 receptor in tumor lymphocytes	5	13	6	1	0	3	0	0
IL6R in interphase zone lymphocytes	3	12	6	5	0	2	0	0
IL6R in tumor cells	1	10	6	7	4	0	0	0

Patient samples (*n* = 28) were scored in a semi-quantitative fashion, reviewed by an expert in pathology (LB) and further transformed into numeric values for statistical analyses according to the following: +++ = 3, ++ (+) = 2.5, ++ = 2, + (+) = 1.5, + = 1, ± = 0.5, = 0.25, and − = 0.0

^a^*n* = 27

Regarding the present IL-6 content of the various tumor-associated cells, the following were determined: intra-tumor lymphocytes: 4/28, interphase zone lymphocytes: 0/28, tumor cells 9/28 and most density was seen in vascular cells: IL6 24/28. In the case of the IL6R, the following numbers were denoted: intra-tumor lymphocytes 4/28, interphase zone lymphocytes: 7/28 and tumor cells: 11/28.

**Fig. 6:**
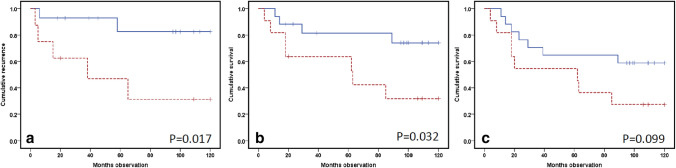
Outcome predictions: **a** recurrence, **b** disease-specific survival, and **c** overall survival of total 28 RCC patients from immunohistochemical quantification of tumor CD3-positive T lymphocytes in surgical resection specimen. The expression levels were qualitatively scored by an experienced pathologist and further dichotomized by median value into high (All patients: *n* = 11/radically treated: *n* = 8) and low (*n* = 17 for all patients/*n* = 14 for radically treated). *p* values come from log-rank tests

The cellular derived measurement did not substantially correlate to tumor diameter or CRP levels. The various above-mentioned variables were also tested regarding prognostic value. In particular, the extent of T lymphocytes (CD3 + cells) infiltration in the tumors predicted survival. A high CD3 + value predicted a decreased survival. This was valid concerning recurrence (*p* = 0.017) and DSS (*p* = 0.032), but not for OS (Fig. 6).

## Discussion

High IL-6 and IL-27 serum levels predicted a worse prognosis. Among clinically presumed radically treated patients, a high IL-6 and high IL-27 strongly predicted a recurrence in both univariate and multivariate analyses. IL-6 also predicted DSS and OS. Overall, the predictions among patients with large tumors (diameter > 7 cm) were excellent regarding both high IL-6 and IL-27 values. Of the soluble receptors studied, high gp130 serum levels predicted a worse OS among the RCC patients with large tumors.

The cytokine levels best predicted survival among patients with large tumors. These findings support that a high cytokine value points to a biologically aggressive tumor, more than a low differentiated tumor [8, 10, 24].

Patients with high levels of IL-6, but only with a clinically localized disease, were more likely to die from their RCC, and also had an increased risk of dying of any cause compared to those with low IL-6 serum values. Our findings are in agreement with those of Ljungberg et al. [25], Hrab et al. [26] and Blay et al. [27].

Increased IL-27 levels predicted recurrence and DSS, especially among patients with a tumor diameter > 7 cm. To the best of our knowledge, this has not been shown in other published studies. Only one study has thus far been published on IL-27 and RCC, which showed that patients with specific polymorphisms of IL-27 are more susceptible to RCC [17].

Soluble IL-6Rα may bind to IL-6, and secondarily bind to gp130 receptors on the surface of cells, in this way stimulating pro-inflammatory functions through *trans*-activation. On the other hand, *cis*-activation is mostly immune inhibitory [28]. We have not shown negative prognostic effects of increased serum IL-6Rα among RCC patients, supporting that IL-6 promotes inflammation in RCC tumors as a mechanism of IL-6-driven carcinogenesis. Soluble gp130 binds the soluble IL-6/IL-6Rα complex [29], and presumably acts as an IL-6/IL-6Rα decoy receptor [28]. We have demonstrated a negative prognostic value of increased gp130 in the serum of patients with larger tumors, which is the opposite of what should be expected. However, gp130 is present on most cells [28], and the increased serum soluble gp130 may be caused by generally increased tumor cellular turnover, which then basically drives the *worse* prognosis.

The combined effect of soluble IL-6Rα, gp130 and IL-6 as to prognosis seems to be complex. Regarding small tumors, the results are as expected, but concerning larger tumors s-gp130 levels paint another picture more consistent with that reflected by s-gp130 levels, e.g., cellular proliferation. Furthermore, the similar survival prediction of IL-6 and IL-27 suggests that this association is limited to cells actually carrying the IL-6 receptor on the surface, as no soluble IL-27 receptor has so far been recognized.

IL-6 and the IL6R may also be determined in tumor tissue [20]. Fu et al. [20] have shown that the expression on tumor cells of IL-6/IL6R worsens the prognosis. We have verified that both the IL-6 and IL6R may be found on cancer cells from RCC patients with high IL-6. Hence, it is supported that IL-6 may act directly on the tumor with a subsequent worse RCC prognosis, both in an autocrine and paracrine manner [30].

We have also shown that among patients with a high IL-6, a surprisingly high expression of IL-6 was found in vascular cells, i.e., endothelial and smooth muscle cells, thereby suggesting that these cells produce IL-6. Endothelial cells are presumably stimulated by VEGF from the tumor [31], with this representing a possible loop where the tumor may become autocrine stimulated.

High IL-6 values in serum also signal a worse OS, and as such, IL-6 values are coupled with many serious diseases [32]. IL-6 is elevated in hypertension, as well as being associated with a higher incidence of future cardiovascular events and mortality [33]. This may partly explain the shown overall survival prediction.

Moreover, we have studied levels of T lymphocyte tumor infiltration and presence in the tumor periphery in a sub-group of patients selected by high IL-6 serum levels. A high T lymphocyte count predicted an increased recurrence and decreased survival. Nevertheless, T regulatory lymphocytes, i.e., Fox P3 lymphocytes, were not to any extent found within the tumor. This is in line with what has previously been shown in head and neck squamous cell carcinomas [34], namely that survival prediction in solid tumors is likely dependent on several immune-related dimensions, like presently one associated with general inflammation through IL-6, and another associated with specific immunity though T lymphocytes [35].

RCC survival prediction is expected to be secondary to factors like the ones included in the Leibovich scores. Leibovich is a composite score, including tumor size, pathological T and N stage, Fuhrman nuclear grading and histological necrosis [36]. With the IL-6 and IL-27 recurrence prediction adjusted by the Leibovich score, both of these cytokine levels still predicted survival.

Clinically, the present results may be relevant. When applying 8 pg/ml IL-6 levels as a cut-off between high and low IL-6 values, 29 patients had high IL-6 values. Six out of nine patients with detectable metastasis at diagnosis had high IL-6 values, as had seven of 14 individuals who subsequently developed RCC metastases. *Several other* patients presumably had other specific causes of their increased IL-6. Of those patients with a low IL-6 who died, or developed recurrent RCC disease (*n* = 10) only one had a RCC tumor with a diameter < 7 cm at diagnosis. The IL-6 values may therefore be utilized at the individual level to sort patients with both a high and low risk of dying because of RCC disease.

Furthermore, ROC analyses suggested that a high IL-27 and IL-6 score predicted a recurrence with both a high sensitivity and specificity, especially as measured in patients with larger tumors. Thus, we have demonstrated that IL-6 and IL-27 may be utilized as biomarkers to identify both a high- and low-risk recurrence of RCC patients at the time of diagnosis.

Patients with high IL-6/IL-27 values at diagnosis may be good candidates for adjuvant treatment with, e.g., VEGF inhibitors [37], as well as with anti-IL-6 therapy such as Siltuximab [38]. The agent Siltuximab (αIL-6) has shown promising results in phase I/II studies for metastatic RCC [38]. It is even possible that a combined blockage of IL-6/IL-27/VEGF would have achieved better results. The results of our study also demonstrate the need for future clinical studies of therapies investigating blockage of gp130 pathways, i.e., bazedoxifene, which blocks p-STAT3 inhibitor [39], and also combined with other blockers like VEGF-TKIs [40] to prolong survival in patients with RCC [41]. However, it should be borne in mind that babies born with a defect gp130 receptor may suffer from extended Stüve-Wiedemann syndrome, which is a serious, often lethal syndrome [42]. Thus, to block gp130 may have serious side effects, making such treatment impossible. Our results also add to knowledge inspiring T cell boosting therapy to be further developed. In any case, the role of IL-27 biology in RCC should be studied judged against the background that new templates for biological therapy in RCC therapy are urgently needed [43].

This study includes a limited number of patients. Therefore, the analyses, especially on the sub-group level showing negative results, must be interpreted with caution. We have measured the cytokines and soluble receptor levels just once. In particular, cytokines in the blood may have a short half-life [44], as a broader picture could have been painted with additional measuring points.

## Conclusions

IL-6 and IL-27 have been shown to have a role in RCC biology through the predictive ability of recurrence and disease-specific survival in otherwise radically treated RCC patients. We believe that patients with a high IL-6 and IL-27 will be good candidates on which to base a biological therapy of RCC. Finally, both these cytokines hold promise for being important in relation to risk stratification regarding RCC prognosis, and thereby a need for treatment.

## Electronic supplementary material

Below is the link to the electronic supplementary material.

Supplementary file1 (DOCX 12 kb)

Supplementary file2 (DOCX 13 kb)

Supplementary file3 (DOCX 12 kb)

## Data Availability

The approval from the ethical committee and informed consent do not cover a full open publication of the dataset. The raw data will be made available in unidentified form on request, and if needed contact the corresponding author.
