# Supplementary material for: Serum levels of the IL-6 family of cytokines predict prognosis in renal cell carcinoma (RCC)
Source: Cancer Immunol Immunother. 2020 Jul 3;70(1):19–30. doi: 10.1007/s00262-020-02655-z (PMC7838134; doi:10.1007/s00262-020-02655-z)
Supplement: Supplementary file 1 — Supplementary file1 (DOCX 12 kb) [file 262_2020_2655_MOESM1_ESM.docx]

Supplementary Table 1: Immunohistochemistry

| Antibody | Source | Epitope retrieved | Dilution | Incubation time (min) | Detection kit |
| --- | --- | --- | --- | --- | --- |
| CD3 (A0452) | DAKO | CC1, 36 min | 1:100 | 32 | UV |
| CD68 (KP1,M0814) | DAKO | CC1, 64 min | 1:5000 | 32 | UV |
| FOXP3 (560044, clone:259D/C7) | BD Biosciences | CC1, 64 min | 1:20 | 32 | UV |
| IL6 (ab 9324) | Abcam | CC1, 48 min | 1:200 | 120 | OV |
| IL6R (ab 128008) | Abcam | CC1, 48 min | 1:800 | 32 | OV |
