# Supplementary material for: Serum levels of the IL-6 family of cytokines predict prognosis in renal cell carcinoma (RCC)
Source: Cancer Immunol Immunother. 2020 Jul 3;70(1):19–30. doi: 10.1007/s00262-020-02655-z (PMC7838134; doi:10.1007/s00262-020-02655-z)
Supplement: Supplementary file 2 — Supplementary file2 (DOCX 13 kb) [file 262_2020_2655_MOESM2_ESM.docx]

Supplementary Table 2: Comparison of tumor characteristics between IL-6 low and high groups (cut-off 8 pg/ml) in those assumed to be radically treated.

| Variable | | All patients  (n=109)(%) | IL-6 low  (n= 86)(%) | | IL-6 high  (n=23)(%) | p-value |
| --- | --- | --- | --- | --- | --- | --- |
| RCC subtypes | |  |  | |  |  |
|  | Clear Cell | 83(76) | 64(74) | | 19(83) | 0.43 |
|  | Papillary | 14(13) | 10(12) | | 4(17) |  |
|  | Chromophobe | 6(5.5) | 6(7) | | 0(0) |  |
|  | Multilocular cystic | 5(4.5) | 5(6) | | 0(0) |  |
|  | Others/ Unclassified | 1(1) | 1(1) | | 0(0) |  |
|  | |  |  | |  |  |
| Size in cm (range) | | 5.3(1.2-18) | 5.0(1.2-18) | | 6.3(1.6--15) | 0.10 |
| pT – Stage^1^ | |  |  | |  |  |
|  | pT1a | 52(48) | 44(52) | | 8(35) | 0.054 |
|  | pT1b | 26(24) | 22(26) | | 4(17.5) |  |
|  | pT2 | 17(16) | 13(15) | | 4(17.5) |  |
|  | pT3 | 10(9) | 5(6) | | 5(21.5) |  |
|  | pT4 | 3(3) | 1(1) | | 2(8.5) |  |
|  |  |  |  | |  |  |
| Nuclear grade^2^ | |  |  | |  |  |
|  | G1-G2 | 62(57) | 51(60) | | 11(48) | 0.71 |
|  |  |  |  | |  |  |
|  | G3-G4 | 46(43) | 34(40) | | 12(52) |  |
|  |  |  |  | |  |  |
| Tumor thrombi | |  | |  |  |  |
|  | Present (n=109) | 6(6) | | 5(6) | 1(4) | 0.78 |
| Positive Margin  Present (n=109) | | 3(3) | | 1(1) | 2(9) | 0.05 |
| Histologic necrosis | |  | |  |  |  |
|  | Present (n=109) | 32(30) | | 23(27) | 9(39) | 0.25 |
| Sarcomatoid components | |  | |  |  |  |
|  | Present (n=109) | 4(4) | | 3(3) | 1(4) | 0.85 |

^1^UICC TNM 2009 version

^2^ 108
