# Supplementary material for: Serum levels of the IL-6 family of cytokines predict prognosis in renal cell carcinoma (RCC)
Source: Cancer Immunol Immunother. 2020 Jul 3;70(1):19–30. doi: 10.1007/s00262-020-02655-z (PMC7838134; doi:10.1007/s00262-020-02655-z)
Supplement: Supplementary file 3 — Supplementary file3 (DOCX 12 kb) [file 262_2020_2655_MOESM3_ESM.docx]

Supplementary Table 3: Cox multivariate regression among patients with recurrence (date) following presumed radical treatment dependent on IL-6 (N=108)/IL-27 (N=91) values adjusted by Leibovich score.

|  | *Hazard ratio* | *95% Confidence Interval* | | *p-value* |
| --- | --- | --- | --- | --- |
|  |  | *Lower* | *Upper* |  |
| **IL-6** | 1.036 | 1.008 | 1.064 | 0.010 |
| **Leibovich group (low vs. intermediate)** | 7.073 | 1.367 | 36.60 | 0.020 |
| **Leibovich group (low vs. high)** | 27.72 | 5.554 | 138.3 | <0.001 |
|  |  |  |  |  |
| **IL-27** | 1.002 | 1.000 | 1.004 | 0.014 |
| **Leibovich group (low vs intermediate)** | 32.20 | 1.681 | 616.6 | 0.021 |
| **Leibovich group (low vs high)** | 103.2 | 6.419 | 1658.0 | 0.001 |
